# Microfluidics of Small-Population Neurons Allows for a Precise Quantification of the Peripheral Axonal Growth State

**DOI:** 10.3389/fncel.2018.00166

**Published:** 2018-06-15

**Authors:** Georg Jocher, Sidney H. Mannschatz, Martin Offterdinger, Rüdiger Schweigreiter

**Affiliations:** Biocenter, Division of Neurobiochemistry, Innsbruck Medical University, Innsbruck, Austria

**Keywords:** microfluidics, sensory neurons, dorsal root ganglia, trigeminal ganglia, neurotrophic factors, leptin, axonal growth state, axonal regeneration

## Abstract

Neurons are morphologically the most complex cell types and are characterized by a significant degree of axonal autonomy as well as having efficient means of communication between axons and neuronal cell bodies. For studying the response to axonal injury, compartmentalized microfluidic chambers (MFCs) have become the method of choice because they allow for the selective treatment of axons, independently of the soma, in a highly controllable and reproducible manner. A major disadvantage of these devices is the relatively large number of neurons needed for seeding, which makes them impractical to use with small-population neurons, such as sensory neurons of the mouse. Here, we describe a simple approach of seeding and culturing neurons in MFCs that allows for a dramatic reduction of neurons required to 10,000 neurons per device. This technique facilitates efficient experiments with small-population neurons in compartmentalized MFCs. We used this experimental setup to determine the intrinsic axonal growth state of adult mouse sensory neurons derived from dorsal root ganglia (DRG) and even trigeminal ganglia (TG). In combination with a newly developed linear Sholl analysis tool, we have examined the axonal growth responses of DRG and TG neurons to various cocktails of neurotrophins, glial cell line-derived neurotrophic factor (GDNF), ciliary neurotrophic factor (CNTF) and leptin. Precise quantification of axonal outgrowth revealed specific differences in the potency of each combination to promote axonal regeneration and to switch neurons into an intrinsic axonal growth state. This novel experimental setup opens the way to practicable microfluidic analyses of neurons that have previously been largely neglected simply due to insufficient numbers, including sensory neurons, sympathetic neurons and motor neurons.

## Introduction

Morphologically, neurons are the most complex cells of the human body. The cell soma extends processes that can measure more than 1 meter in length. Such macroscopic dimensions at the cellular level resulted in axonal growth cones having a significant degree of autonomy and led to the evolution of an intricate bidirectional signaling network between axonal termini and cell bodies (Kandel, 2013). Both the local autonomy of growth cones and efficient axonal communication are essential features for axonal regeneration after injury (Navarro et al., 2007; Bosse, 2012). Because neuronal cell bodies are not accessible to therapeutic intervention after nerve injury, any regeneration promoting treatment regimens have to target severed nerve fibers directly. From a clinical viewpoint it is therefore of little value to carry out experiments on axonal regeneration without distinguishing between axons and cell bodies. To achieve just that, a compartmentalized neuronal culture system was introduced that fluidically segregated axons from cell bodies (Campenot, 1977). This experimental setup, which became known as the Campenot chamber, allowed for the treatment of axons independently from somas. Practical shortcomings, however, prevented widespread use of this culture system. It was only with the adoption of microlithographic techniques in life sciences that compartmentalized neuronal cultures could be generated in a highly controllable and reproducible manner (Neto et al., 2016). The design of the original microfabricated device presented by Taylor et al. (2005) has become a standard in the field of microfluidic cell culture, particularly in neuroscience. Interestingly, while the Campenot chamber has, for technical reasons, been largely restricted to neurons of the peripheral nervous system (PNS), modern microfluidic devices have primarily focused on neurons of the central nervous system (CNS). Microfluidic chambers (MFCs) require a significantly larger number of neurons for seeding than Campenot chambers. This drawback has hindered their use for investigations into peripheral neurons, which are generally much less abundant than CNS neurons. Half a million neurons are typically required to load one MFC (Tsantoulas et al., 2013; Jia et al., 2016, 2018), and while this large number is not an issue for typical CNS neurons, it is a challenge for neurons of the PNS, especially when working with PNS neurons from adult mice. Per mouse, for instance, one can obtain at most 100,000 sensory neurons from dorsal root ganglia (DRG; Heinrich et al., 2016), and significantly fewer still from trigeminal ganglia (TG; Katzenell et al., 2017). Performing a series of experiments with such small-population neurons in MFC devices is therefore impractical, if not unfeasible. To overcome this bottleneck, we have established a simple MFC protocol that allows for a dramatic reduction in the number of required neurons to only 10,000, which equals one adult mouse per microfluidic chamber when working with TG neurons, and one adult mouse for a number of chambers when dealing with DRG neurons. This advanced microfluidic protocol, along with a novel linear Sholl analysis tool, not only markedly reduces the consumption of lab animals, but also facilitates microfluidic experiments with small-population neurons at an unprecedented speed and efficiency. We used this novel approach to precisely quantify the intrinsic axonal growth state of DRG and TG neurons from adult mice as induced by topical application of neurotrophic factors and neuropoietic cytokines to the axonal compartment. The results obtained show remarkably little experimental variability and reveal specific differences in the potency of various growth factor combinations to switch neurons into an axonal growth state.

## Materials and Methods

### Animals

Sensory neurons were obtained from adult (2–6 months of age) C57Bl/6N mice. All experimental protocols were approved by the Austrian Animal Experimentation Ethics Board in compliance with the European Convention for the Protection of Vertebrate Animals Used for Experimental and other Scientific Purposes (ETS no. 123).

### Preparation of Neurons From Dorsal Root Ganglia and Trigeminal Ganglia

DRG were prepared as described previously (Eckharter et al., 2015). Briefly, DRG from all segmental levels were collected and incubated twice with Liberase DL (Roche # 05401160001; 0.25 mg/ml) for 35 min at 37°C. After rinsing with PBS, DRG were incubated with 0.05% Trypsin-EDTA (Sigma #T3924) for 28 min at 37°C. DRG were rinsed again in PBS and triturated in 1.5 ml of neuronal culture media TNB-100 (Biochrom #F8023; supplemented with the cocktail #F8820, glutamine and penicillin/streptomycin) using a fire-polished Pasteur pipette. The neuronal suspension was loaded on top of 7.5 ml of 3.5% BSA (Sigma #A7906) dissolved in DMEM and centrifuged at 14× *g* for 15 min. After centrifugation, the neuronal pellet was resuspended in 1 ml of TNB-100 media, and neurons were counted in a Neubauer chamber. The density was adjusted to 2000 neurons per μl using neuronal culture media.

TG neurons were prepared as described previously (Malin et al., 2007). Briefly, TG were incubated for 20 min at 37°C with 40 U/ml papain (Worthington #3126) dissolved in HBSS containing 0.75 mg/ml L-cysteine and 2 μl/ml saturated NaHCO_3_, before incubating with collagenase type II (Worthington #4176; 4 mg/ml) and dispase type II (Sigma #D4693; 4.7 mg/ml), dissolved in HBSS, for 20 min at 37°C. Ganglia were triturated in 0.5 ml neuronal culture media using a fire-polished Pasteur pipette and loaded on top of a Percoll gradient consisting of 12.5% and 28% fractions. After centrifugation at 1300× *g* for 10 min, the neuronal pellet was resuspended in 0.25 ml of TNB-100 media, and neurons were counted in a Neubauer chamber. The density was adjusted to 2000 neurons per μl using neuronal culture media. After 3 days *in vitro* (at DIV3) the media in the somatic compartment was supplemented with 2.5 μg/ml cytosine arabinoside (Sigma #C1768) to block the proliferation of non-neuronal cells.

### Assembling the Microfluidic Device

For generating compartmentalized neuronal cultures, we used the tripartite microfluidic chamber from Xona Microfluidics with 500 μm long microgrooves (#TCND500). Neuronal cell bodies were seeded into the central compartment from where they extended processes into the left and right axonal compartments. The MFC was bonded onto a custom-made cover glass (Assistant; φ = 50 mm; 0.17 ± 0.01 mm). Cover glasses were cleaned in concentrated nitric acid (65%) for 18–36 h on a horizontal shaker. Nitric acid was replaced with dH_2_O and cover glasses were rinsed for at least 48 h with repeated exchanging of dH_2_O, and autoclaved dH_2_O used for the final rinse. Cover glasses were left to dry for 1 h in a biosafety cabinet before being exposed to UV light for 10 min. Subsequently, they were coated with poly-L-lysine (PLL; Sigma #P4707; 0.01%) overnight at 37°C in an 8.5% CO_2_ incubator. Right before use, PLL was aspirated and the cover glass was washed three times with sterile dH_2_O and left to dry for 45 min. The MFC was sterilized by incubation in 70% ethanol for 5 min. After rinsing extensively with sterile dH_2_O, it was left to dry for 45 min. For bonding, the MFC was evenly placed on top of the PLL-coated cover glass, and cautiously pressed down with forceps. After successful bonding, the assembled device was placed on top of an ice-cold metal block (equilibrated at −20°C for 10 min) and then incubated for 5 min at 37° C in order to induce condensation, which eases flooding of the microgrooves with laminin. After that, 300 μl of laminin (Sigma #L2020), diluted to 20 μg/ml in HBSS, was added into the top central well of the somatic compartment. When all microgrooves were flooded with laminin, 100 μl of laminin was added into the top central well of both axonal compartments. The MFC was incubated for 2 h at 37°C in an 8.5% CO_2_ cell culture incubator. After coating, the MFC was rinsed with HBSS three times for 10 min, and finally with TNB-100 media. Incubation with neuronal culture media was always for at least 1.5 h to allow for the proper equilibration of microgrooves. We tested plasma bonding by using the glow discharge option of a BenchTop Turbo from Denton Vacuum. Both cover glasses and MFCs were exposed to nitrogen plasma in a vacuum between 300 and 500 millitorr over a time period of 1–5 min.

### Matrigel Barrier

Before loading DRG neurons into the MFC, a Matrigel barrier was placed in the bottom well, which did not seal the main channel, but kept the neuron suspension within the main channel’s exit area. Thus, air can escape and the neuronal suspension can move along the main channel towards the bottom well. After aspiration of TNB-100 media from all three compartments of the MFC, the main channel was rinsed once with sterile dH_2_O in order to get rid of salts. All liquid was removed from the main channel and the bottom well by vacuum aspiration. Ten microliter Matrigel (Corning #354230) were pipetted into the lower part of the bottom well. After 8–10 min of gelling at room temperature (RT), the Matrigel was rearranged with a pipette tip such that a semicircle around the exit of the main channel in the upper part of the bottom well was formed. After 1 min, the Matrigel was fully gelled and the MFC was ready for loading with neurons.

### Loading of Neurons Into the MFC

Neurons were loaded within a time window of 11–14 min following the aspiration of all liquid from the MFC. The volumetric capacity of the main channel was approximately 5 μl, and this volume was used for loading. The density of the neuronal suspension was 2000 neurons per μl, thus a total number of 10,000 neurons were seeded into an MFC. The neuron suspension was loaded into the top well of the somatic compartment from where it moved towards the bottom well but was ultimately stopped by the Matrigel barrier. Immediately after seeding, 30 μl of TNB-100 media was added to the top wells of the axonal compartments. The MFC was put into an 8.5% CO_2_ incubator and neurons were allowed to attach for 2–3 h. Finally, 60 μl of TNB-100 media was added to the top well of the somatic compartment. The Matrigel barrier usually dissolves by itself overnight.

### Application of Neurotrophic Factors and Neuropoietic Cytokines

The day after seeding (at DIV1), the media was removed and 140 μl of TNB-100 was added to the somatic compartment. The axonal compartments were flooded with 100 μl TNB-100 containing a selection of neurotrophic factors or neuropoietic cytokines specified as followed: 50 ng/ml nerve growth factor (NGF; Stem Cell Technologies #78092), 50 ng/ml brain-derived neurotrophic factor (BDNF; Stem Cell Technologies #78005), 50 ng/ml neurotrophin 3 (NT-3; Stem Cell Technologies #78034), 50 ng/ml glial cell line-derived neurotrophic factor (GDNF; Stem Cell Technologies #78058), 20 ng/ml ciliary neurotrophic factor (CNTF; Stem Cell Technologies #78010), and 50 ng/ml leptin (Sigma #L3772). The gradient was renewed after 48 h (at DIV3) by removing the media in the compartments and refilling them as described above.

### *In Vitro* Axotomy

At DIV5, an *in vitro* axotomy (IVA) was performed by entirely filling the wells of the axonal compartment with media and suctioning off all liquid by vacuum aspiration alternately in the top and the bottom well with the volume of the whole well suctioned through the axonal compartment. After 2–3 rounds, the axonal compartment was devoid of axons and debris. One-hundred and forty microliter of fresh TNB-100 media was added to the somatic compartment, and 100 μl TNB-100 media containing neurotrophic factors or neuropoietic cytokines was added to the axonal compartments. Neurons were fixed 23 h after axotomy.

### Immunocytochemistry

Neurons were fixed with 4% PFA. All three top wells were filled with PFA (up to approx. 300 μl) and incubated for 10 min at RT. The liquid was removed from all wells and the wells of the axonal compartments were filled again with PFA. Only 100 μl of PFA were added to the top and the bottom well of the somatic compartment to facilitate liquid flow from the axonal to the somatic compartment thereby ensuring fixation of axons located in the microgrooves. After incubating for another 10 min, the MFC was washed three times for 10 min with 1× PBS. Blocking solution (3% NGS, 1% BSA, 0.1% Triton X-100, in PBS) was added to the wells (300 μl to the axonal top wells, and 100 μl to the somatic top well) and incubated at RT for 2 h before primary antibodies were added as indicated: Tuj1 (mouse anti-ß3 tubulin; Sigma #T8578; 1:1000) into the axonal compartments and NeuN (mouse anti-neurofilament; Chemicon #MAB377; 1:500) into the somatic compartment. Primary antibodies were incubated overnight at 4°C, followed by four 10-min washes with 1× PBS and exposure to the secondary antibodies for 4 h at RT. Secondary antibodies used were goat anti-mouse Alexa 488 (Life Technologies #A-11029) and goat anti-mouse Alexa 555 (Life Technologies #A32727). After staining with the secondary antibodies, the main channel was rinsed with 1× PBS containing Hoechst (1:10,000) for 1 min. Finally, the somatic and the axonal compartments were washed three times for 10 min with 1× PBS.

### Imaging

A Leica DMi8 inverted wide-field microscope was used to image neurons and axons in the MFC system. An image of the entire MFC (main channel and the two axonal compartments) was recorded with a HC PL FLOUTAR 10×/0.3 dry objective in two channels: Alexa 488 was imaged using the 470 nm LED of a SpectraX LED light source in the first channel. A phase contrast image was acquired in the second channel. The whole chamber was imaged by performing a tilescan of 5 × 7 single images that were merged by auto-stitching with 15% overlap and smooth blending using Leica’s LAS X software. Another tilescan (3 × 5 images, settings as above) was carried out in order to image the neuronal cell bodies present in the main channel by recording three channels (NeuN-Alexa 555, Hoechst 395, phase contrast).

### Image Processing

We have generated custom-designed MATLAB scripts in order to rotate the images of the chambers such that the chamber (as seen in the phase contrast image) is aligned exactly along the image axes. Technically this was achieved by a Hough-transformation thus specifying the rotation angle in the phase contrast image. We also used the Hough-transformation to identify the boundaries of the left and right analysis zones. Outputs of this first script were rotated and cropped fluorescence and phase contrast images (left and right axonal compartments). The second script implemented a linear Sholl analysis by identifying the Alexa 488 positive axons using an adaptive thresholding strategy. The obtained masks (left and right axonal compartments) were binarized, skeletonized and the number of positive pixels along vertical grid lines drawn at 50 μm intervals was calculated and plotted against the distance from the microgrooves. Finally, data were normalized against the number of NeuN positive neurons in the main channel. Both scripts are available upon request.

### Data Analysis and Statistics

Statistical analysis was performed using SPSS 24.0 (IBM). Specifically, a two-way repeated-measures analysis of variance (ANOVA) was performed for each experimental set. The results were reported as mean ± SEM for at least three independent experiments. A *p*-value of ≤0.05 was considered statistically significant.

## Results

### A Dramatic Reduction in the Number of Neurons Required for Loading an MFC

**Figure 1:**
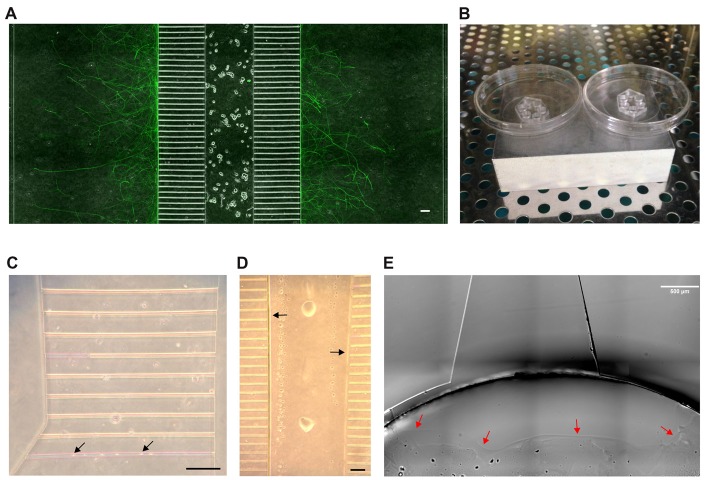
The microfluidic setup. **(A)** Section of a tripartite microfluidic chamber. Adult dorsal root ganglia (DRG) neurons have been seeded into the central main channel from where they extend axons bidirectionally into the axonal compartments through the 500 μm long microgrooves. A composite image of phase contrast and Tuj1 staining is shown. Scale bar is 100 μm. **(B)** The assembled microfluidic devices are placed on top of an ice-cold metal block and put into an incubator at 37°C to induce condensation. **(C)** The flooding of microgrooves with laminin is eased by condensation. Microgrooves appear pink before flooding. Black arrows indicate droplet formation. Scale bar is 100 μm. **(D)** By 11–14 min after aspiration, nearly all liquid has evaporated from the main channel except for some droplets and a thin liquid border along the edge of the main channel (black arrows). This border eases the loading of the main channel with neurons. Scale bar is 100 μm. **(E)** A Matrigel barrier is placed in the bottom well around the exit from the main channel to prevent the neuronal suspension from occupying the bottom well (red arrows). Scale bar is 500 μm.

The yield of DRG neurons per adult mouse is limited to at most 100,000 neurons (Heinrich et al., 2016), but the numbers indicated are often significantly lower; see, for instance, (Lee and Levine, 2015). We ourselves manage to dissect 40–50 DRG per mouse (out of 62 ganglia in total), which typically does not result in more than about 50,000 neurons. From the two TG per animal we obtain at most 10,000 neurons. Such a yield is poor when related to the number of neurons required to load an MFC, the standard reference of which is the MFC device manufactured by Xona Microfluidics and described by Taylor et al. (2005). Throughout this study we used Xona’s tripartite chamber (Figure 1A), but the dual chamber can be used as well. The number of neurons required per MFC varies and presumably depends on the experimenter’s experience with handling microfluidic devices, but based on the existing literature approximately 500,000 DRG neurons are required per MFC (Tsantoulas et al., 2013; Jia et al., 2016, 2018). The reason for this large number is that, first, the chamber has to be loaded with a suspension of very high density of several million neurons per milliliter otherwise too few neurons will adhere in the main channel. Second, a suspension volume of at least 25–50 μl has to be applied to the upper well to allow for the suspension to flow through the main channel into the bottom well, especially when using non-plasma bonded devices. Importantly, for neurons to attach in the main channel the flow has to cease, which is accomplished when the liquid reaches a state of equilibrium between the top and bottom well. To overcome this bottleneck for small-population neurons, such as DRG and TG neurons of adult mice, we have developed a novel microfluidic strategy that does not require more than 10,000 neurons per MFC.

To start with, we tested both plasma and non-plasma bonded devices and found that, while the seeding of neurons into a plasma bonded device is much easier than without plasma bonding, sensory neurons tend to cluster in the main channels of plasma bonded devices. Clustering is disadvantageous because it interferes with the counting of neurons and results in a pattern of axonal outgrowth that is different from the axonal outgrowth shown by neurons cultured in a well-balanced network, which makes any comparisons invalid. We therefore opted for non-plasma bonding for this study, but the protocol presented herein is equally applicable to plasma bonded devices. The central disadvantage of non-plasma bonding is that the surfaces are significantly less hydrophilic than with plasma bonding, which hampers coating and seeding. It therefore would be preferable to bond the MFC onto a glass coverslip that has already been pre-coated with PLL and laminin. In such a setup, however, the bond between the device and the cover glass is not sufficiently tight, typically resulting in leakage and growth of axons outside the microgrooves. We therefore bonded the MFC onto a PLL-coated cover glass and subsequently added laminin. To ensure the proper coating of all compartments with laminin, including the microgrooves, we made use of condensation. Condensation greatly facilitates flooding of all compartments and microgrooves with laminin (Figures 1B,C). Relying on condensation within the MFC to facilitate flooding with laminin is also relevant when dealing with plasma-bonded devices, because the hydrophilic surfaces persist for only a very limited time period. We observed that the hydrophilic effect was completely gone by 48 h after the glow discharge, which prevents any long-term storage of plasma bonded neuronal devices. Overcoming hydrophobicity is also an issue when loading neurons into the MFC. Triggering condensation is not an option when dealing with a neuronal suspension, but we observed that there is a short time window within which the seeding of neurons is greatly facilitated. Specifically, we determined that the time window between 11 min and 14 min after aspirating off all the liquid from the MFC to be best for loading neurons into the chamber. It is within this time window that a liquid border persists along the chamber’s internal surfaces that significantly facilitates flooding of the main channel with the neuronal suspension (Figure 1D). Loading before 11 min is likely to result in a “liquid short” between the inflowing suspension and the liquid still present, and the subsequent formation of an air bubble within the main channel that prevents any further movement of the suspension. After a waiting time of more than 14 min prior to loading the MFC, the internal moisture is reduced to such an extent that capillary forces do no longer support the flow of the suspension into the main channel. Through extensive pipetting it might be possible to squeeze the suspension into the MFC, but this impairs the viability of neurons and increases the risk of leakage. For loading we used a suspension volume of merely 5 μl, which roughly equals the volume of the main channel, containing 10,000 neurons. In principle, diluting the suspension could further reduce the number of neurons, but we found that 10,000 neurons give a reasonable density in the main channel. To prevent the suspension from entering the bottom well after passing through the main channel, which would not only result in a waste of neurons but also in a backlash flow towards the top well, we placed a Matrigel barrier in the bottom well right around the exit from the main channel (Figure 1E) thereby focusing the distribution of neurons to the main channel. Importantly, to achieve such high seeding efficiency, the main channel has to be empty before loading the neurons. After allowing neurons to attach for 2–3 h, all wells were filled with media.

**Figure 2:**
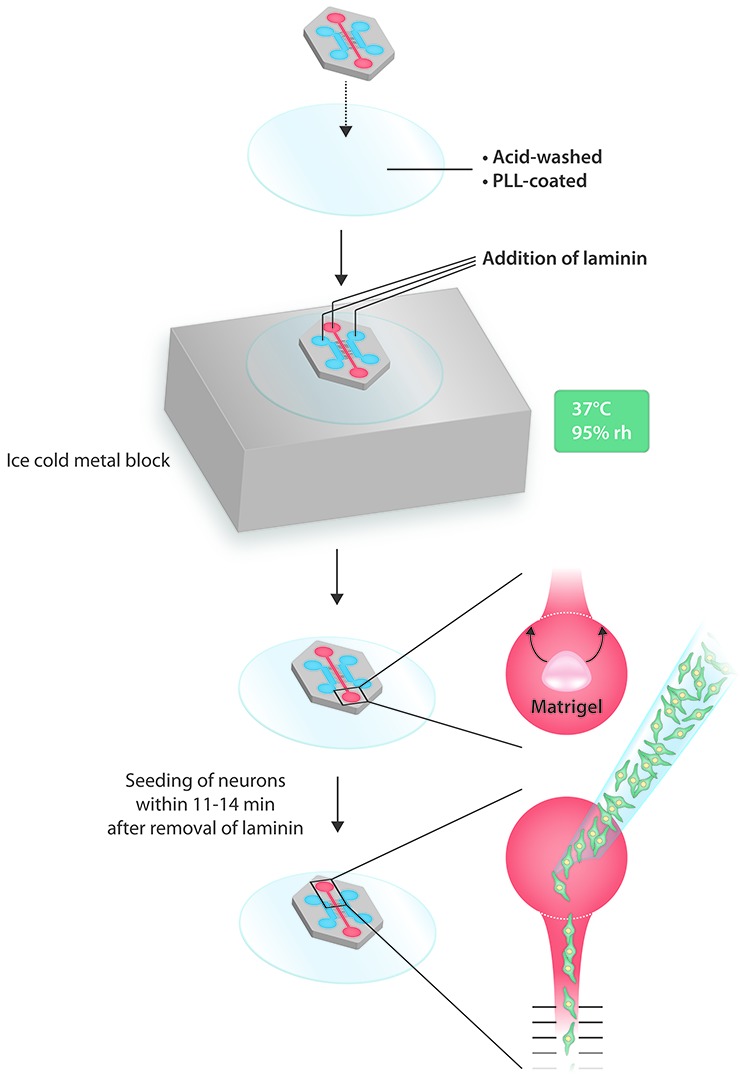
Schematic summary of the microfluidic procedure.

In summary, this is a simple and reliable procedure for efficiently seeding an MFC with as little as 10,000 neurons (Figure 2).

### A Linear Sholl Analysis Tool for Analyzing Axonal Growth Within the MFC

**Figure 3:**
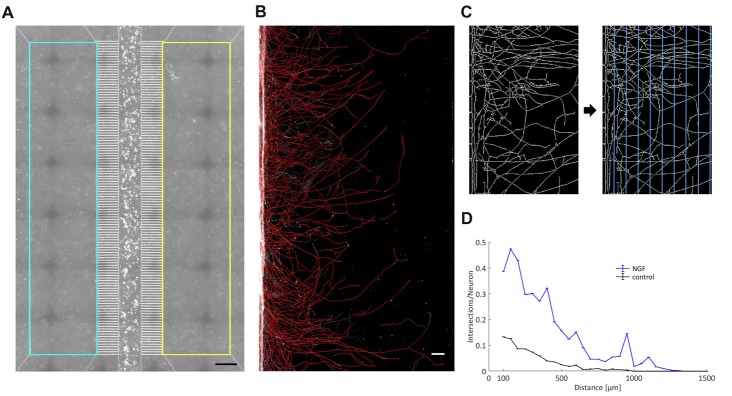
A linear Sholl analysis tool. **(A)** By employing the chamber’s phase contrast image, two rectangles comprising the two axonal compartments have been defined as region of interest (ROI) within which the fluorescence signal of the axonal processes was analyzed. Scale bar is 500 μm. **(B)** A detailed view into an axonal compartment. Binarized and skeletonized processes are shown in red and are superimposed on the original black-and-white fluorescent image. Scale bar is 100 μm. **(C)** Magnification of skeletonized axons. Vertical grid lines with a spacing of 50 μm were laid upon the skeletonized axons. The number of axonal intersections was determined for each line. **(D)** Readout of a single experiment with the two curves representing the two axonal compartments. The number of intersections was normalized by the total number of neurons in the main channel and plotted against the width of an axonal compartment. “0” denotes the exit from the microgrooves.

Axonal outgrowth of individual neurons *in vitro* is typically measured through a circular Sholl analysis, and free software tools that accomplish this task have been made available (Rishal et al., 2013). To measure axonal growth in microfluidic devices, axonal compartments have to be geometrically aligned and defined as region of interest (ROI). Within that ROI, immunofluorescent signals from axons have to be binarized and skeletonized, vertical grid lines drawn with a defined spacing, and the number of axonal intersections counted on each such line. To our knowledge there is no accessible software tool that does just that. For this reason, we have written two MATLAB scripts that carry out a linear Sholl analysis of axons grown in the compartments of an MFC (Figures 3A–D). The readout provides the number of axonal intersections per grid line in relation to the number of neurons in the main channel and is plotted against the distance from the microgrooves (Figure 3D). We spaced the grid lines in 50 μm increments. Because the axonal density is particularly high at microgrooves exits, and because axonal debris often accumulates there following IVA, we omitted the measurement at the first grid line at 50 μm and started to measure at 100 μm from the microgrooves.

### Promoting Primary Axonal Outgrowth With Cocktails of Neurotrophic Factors and Neuropoietic Cytokines

We made use of this novel microfluidic strategy to evaluate the effects of topically applied neurotrophic factors and neuropoietic cytokines on axonal outgrowth and regeneration of sensory neurons derived from adult mice. Specifically, we added three groups of neurotrophic factors and neuropoietic cytokines into the axonal compartment: first NGF only, second NGF, BDNF and NT-3 (referred to as 3x cocktail), and third NGF, BDNF, NT-3, GDNF, CNTF and leptin (referred to as 6x cocktail). While the role of the neurotrophins as well as GDNF in promoting axonal growth has already been well documented (Markus et al., 2002; Tucker, 2002), the respective function of CNTF, and in particular leptin, remains to be fully addressed.

**Figure 4:**
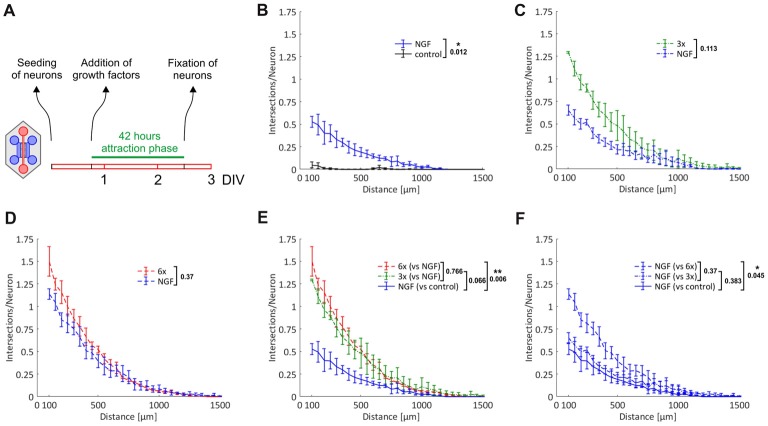
Chemoattraction and promotion of primary axonal outgrowth by neurotrophic factors and neuropoietic cytokines. Axons from adult DRG neurons were attracted by a selection of neurotrophic factors and neuropoietic cytokines for 42 h. Axonal growth in the two axonal compartments was quantified by linear Sholl analysis. **(A)** Experimental time course. **(B)** Nerve growth factor (NGF) vs. control; *n* = 3. **(C)** 3x cocktail vs. NGF; *n* = 3. **(D)** 6x cocktail vs. NGF; *n* = 3. **(E)** Combination of treatment curves from **(B–D)**: NGF, 3x cocktail, 6x cocktail. **(F)** Combination of NGF curves from **(B–D)**: NGF (vs. control), NGF (vs. 3x cocktail), NGF (vs. 6x cocktail). 3x cocktail: NGF, brain-derived neurotrophic factor (BDNF), neurotrophin 3 (NT-3); 6x cocktail: 3x cocktail plus glial cell line-derived neurotrophic factor (GDNF), ciliary neurotrophic factor (CNTF), leptin. Data represent mean ± SEM for each increment; *p* values are indicated; *p ≤ 0.05, **p ≤ 0.01.

To start with, we performed simple primary outgrowth experiments with DRG neurons on permissive laminin substrate. At DIV1, growth factors were added to the axonal compartment and cultures were fixed after 42 h, when the first pioneering axons began to approach the compartment boundary lying opposite to the microgrooves (Figure 4A). As shown in Figures 4B–D, the gradual enrichment of trophic factor cocktails in the axonal compartment gradually increased total axonal outgrowth. There was a clear trend towards an increased outgrowth when supplementing NGF with BDNF and NT-3 (3x cocktail) although it did not quite reach statistical significance (*p* = 0.066). The 6x cocktail, however, did lead to a significant increase from NGF alone (*p* = 0.006), with a small further outgrowth increase from the 3x cocktail (Figure 4E). Interestingly, the axonal responsiveness to NGF was significantly enhanced when the 6x cocktail had been added to the opposite axonal compartment (*p* = 0.045; Figure 4F). Evidently, neurons stimulated with the 6x cocktail in one axonal compartment switch into an axonal growth state that is translated to the NGF compartment. In the absence of any growth factor, hardly any axon makes it into the axonal compartment within the given time interval, highlighting the chemoattractive role of growth factors in this experimental setup.

### Growth Response to Neurotrophic Factors and Neuropoietic Cytokines Following *in Vitro* Axotomy

**Figure 5:**
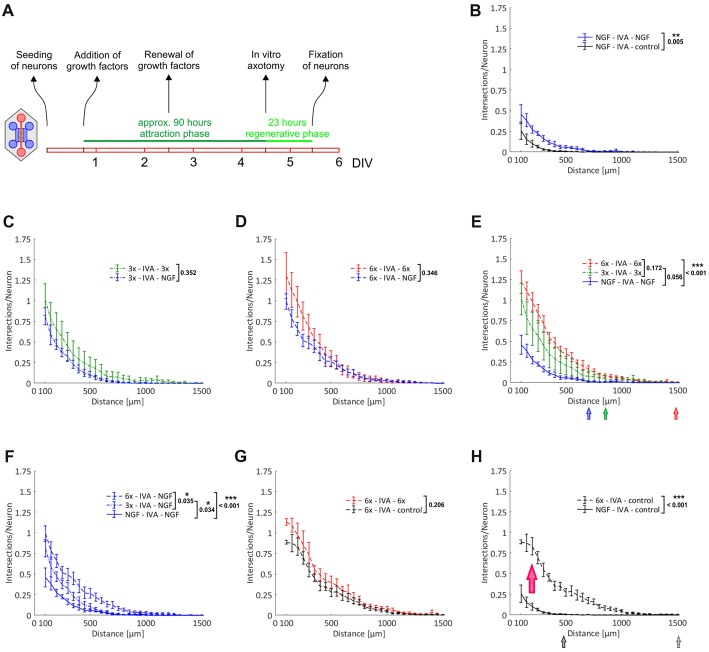
Induction of an axonal growth state in adult DRG neurons. Axons from adult DRG neurons were attracted over nearly 4 days by a selection of neurotrophic factors and neuropoietic cytokines. After *in vitro* axotomy (IVA), axons were allowed to regenerate for 23 h in the presence or absence of a selection of neurotrophic factors and neuropoietic cytokines. Axonal growth in the two axonal compartments during this regenerative period was quantified by linear Sholl analysis. **(A)** Experimental time course. **(B)** Attraction with NGF and regeneration with NGF vs. control; *n* = 8. **(C)** Attraction with 3x cocktail and regeneration with 3x cocktail vs. NGF; *n* = 5. **(D)** Attraction with 6x cocktail and regeneration with 6x cocktail vs. NGF; *n* = 5. **(E)** Combination of treatment curves from **(B–D)**: NGF, 3x cocktail, 6x cocktail (pooled data from **D** and **G**). The median values of the outermost grid lines with axonal intersections are indicated for each treatment. **(F)** Combination of NGF curves from **(B–D)**: NGF (vs. control), NGF (vs. 3x cocktail), NGF (vs. 6x cocktail). **(G)** Attraction with 6x cocktail and regeneration with 6x cocktail vs. control; *n* = 5. **(H)** Combination of control curves from **(B,G)**: control (vs. NGF), control (vs. 6x cocktail). The red arrow indicates the shift of neurons into an axonal growth state. The median values of the outermost grid lines with axonal intersections are indicated for each control. 3x cocktail: NGF, BDNF, NT-3; 6x cocktail: 3x cocktail plus GDNF, CNTF, leptin. Data represent mean ± SEM for each increment; *p* values are indicated; *p ≤ 0.05, **p ≤ 0.01, ***p ≤ 0.001.

The primary outgrowth paradigm does not distinguish between the chemoattraction of axons and direct stimulation of axonal growth without a chemoattractive aspect. To allow for that distinction, we axotomized neurons in the axonal compartment after an initial attraction phase by vacuum aspiration. In this setup, all axons are lined up in the exits from the microgrooves and are ready to respond to stimuli. Not only is chemoattraction no longer required as a prerequisite for axonal growth to occur in the axonal compartment, but all axons now have the same starting line, which we expected would lead to a noticeable reduction of data variability. After promoting axonal growth over a period of nearly 4 days, which resulted in the formation of a dense axonal meshwork in the axonal compartment (data not shown), neurons were axotomized. Cultures were fixed following a regenerative growth phase over 23 h, when the first pioneering axons began to approach the compartment boundary lying opposite to the microgrooves (Figure 5A). As shown in Figures 5B–E, there was a clear trend towards an increased outgrowth when stimulating with the 3x cocktail in comparison to NGF only, although it did not quite reach statistical significance (*p* = 0.056). The 6x cocktail, however, did lead to a significant increase from NGF alone (*p* < 0.001), with a small further outgrowth increase from the 3x cocktail, although the difference between the 3x and 6x cocktail was not statistically significant (Figure 5E). Importantly, the gradual enrichment of trophic factor cocktails also promoted the elongation of pioneering axons, as documented with a shift of the outmost grid lines still harboring axonal intersections to greater distances from the microgrooves (Figure 5E). Interestingly, the response to NGF seemed to be linked to the kind of cocktail used in the attraction phase. Specifically, the more trophic factors were used for attraction, the more potent was the growth-stimulating effect of NGF in the regenerative phase (Figure 5F). When we attracted axons with the 6x cocktail and did not add any growth factor during the regenerative phase, however, we observed a regenerative response that was as potent as when adding NGF (Figure 5G vs. Figure 5D). Therefore, the regenerative response was not caused by the acute treatment with NGF but was due to the previous treatment with the growth factor cocktail. Evidently, neurons switched into an axonal growth state during the nearly 4 days of continuous treatment with growth factors and cytokines and exhibited enhanced axonal growth during the regenerative phase irrespective of any acute treatment (*p* < 0.001; Figure 5H). Even without any further treatment during the regenerative phase, pioneering axons were more than three times the length in neurons that had been primed with the 6x cocktail than when they had been stimulated with NGF only, as illustrated by the respective median position of the outmost grid lines still harboring axonal intersections.

In summary, the 6x cocktail proved to be more potent in inducing such an intrinsic axonal growth state than the 3x cocktail or NGF only, and it will be very interesting to explore this priming effect in more detail in future studies.

### A Comparison of Axonal Regeneration of DRG- and TG-Derived Sensory Neurons

**Figure 6:**
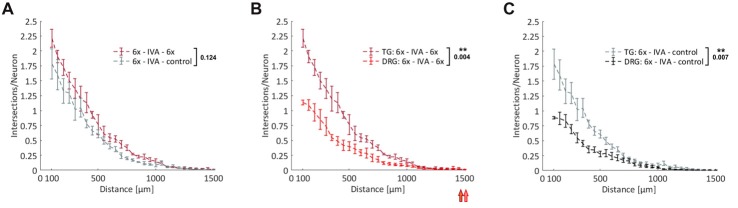
Comparison of axonal regeneration of DRG- and trigeminal ganglia (TG)-derived sensory neurons. **(A)** Axons from adult TG neurons were attracted over nearly 4 days by the 6x cocktail. After IVA, axons were allowed to regenerate for 23 h in the presence or absence of the 6x cocktail. Axonal growth in the two axonal compartments during this regenerative period was quantified by linear Sholl analysis; *n* = 5. **(B)** Combination of treatment curves from **(A)** and Figure 5G: 6x cocktails. The median values of the outermost grid lines with axonal intersections are indicated for the two curves. **(C)** Combination of control curves from **(A)** and Figure 5G (control). 3x cocktail: NGF, BDNF, NT-3; 6x cocktail: 3x cocktail plus GDNF, CNTF, leptin. Data represent mean ± SEM for each increment; *p* values are indicated; **p ≤ 0.01.

Axonal regeneration studies with DRG-derived sensory neurons have potential clinical relevance for a host of peripheral nerve lesions and using our novel experimental setup we managed to load up to five MFCs with DRG neurons obtained from a single adult mouse. Cranial ganglia, however, are even more restrictive in terms of the number of neurons that can be obtained for experiments, but nevertheless they are highly relevant clinically, as exemplified by studies on neuropathic pain (Iyengar et al., 2017). From the two TG per mouse, we were able to obtain about 10,000 neurons, which was just sufficient to load one MFC. Since sensory neurons derived from DRG and cranial ganglia are partially of different embryonic origin (Lindsay et al., 1985), we were curious to compare the axonal growth response of DRG- and TG-derived neurons within the experimental paradigm described above. We attracted neurons with the 6x cocktail and compared the regenerative response of neurons with continued treatment of the 6x cocktail during the regenerative phase to those no longer treated with any growth factors. As with DRG-derived neurons, we observed a remarkable priming effect such that neurons without acute treatment during the regenerative phase regenerated nearly as well as neurons treated with the 6x cocktail (Figure 6A). When we compared these two types of sensory neurons, treated under the same conditions, we found there to be a consistently stronger total axonal outgrowth in TG-derived neurons than DRG-derived neurons (*p* = 0.004 for 6x cocktail and *p* = 0.007 for control; Figures 6B,C). Specifically, the number of intersections at the exit of the microgrooves was twice as high with TG neurons than with DRG neurons indicating a larger fraction of process-bearing TG neurons and/or a higher branching index of the neurons. The growth of pioneering axons, however, did not differ between these two types of sensory neurons, as indicated by the respective median position of the outmost grid lines still harboring axonal intersections.

Taken together, this comparison of two different types of adult sensory neuron populations revealed a similar axonal responsiveness to neurotrophic factors and cytokines, but at the same time demonstrated different basic modes of axonal outgrowth in terms of the mean number of processes per neuron.

## Discussion

We present here a novel experimental approach that greatly facilitates the use of microfluidic devices for the study of small-population neurons. Specifically, we describe how the number of neurons required for seeding a single microfluidic device can be reduced from several hundred thousand to only 10,000 neurons. This procedure allows for efficient microfluidic experiments with DRG-derived neurons from adult mice, and even with neurons derived from cranial ganglia. Employing this novel experimental strategy, we were able to demonstrate and precisely quantify the intrinsic axonal growth state of regenerative sensory neurons from adult mice.

The key elements of this novel strategy are, first, that the neuron suspension is loaded into an empty main channel and, second, that the suspension is prevented from spreading into the bottom well by placing a Matrigel barrier around the exit of the main channel. This is in explicit contrast to established protocols, which rely on loading the neuronal suspension into a fully flooded main channel and allowing the suspension to establish fluidic balance between the top and bottom well (Taylor et al., 2005; Park et al., 2006). The central problem that arises when seeding into an empty main channel is the lack of capillary forces, especially when using non-plasma bonded MFCs. We observed, however, that there is a time window between 11 min and 14 min after aspirating all liquid off from the MFC that allows for the convenient loading of neurons into the chamber. During this time window a liquid border persists along the chamber’s internal surfaces, which greatly eases flooding of the main channel with the neuronal suspension. The lack of capillary forces is also an issue when loading the MFC with laminin. We have solved this problem by inducing condensation within the MFC prior to the addition of laminin. While these measures are primarily aimed for non-plasma bonded devices, which are the method of choice for laboratories lacking a plasma cleaner, we have observed that they are equally useful when employing plasma bonded MFCs, in particular when using stored chambers that have lost some of their super-hydrophilicity. The key advantage of plasma bonded devices is the greatly facilitated flooding of the main channel and microgrooves with the neuronal suspension and coating material, respectively. We do confirm this, but at the same time we have observed a persistent tendency of sensory neurons to aggregate in plasma bonded MFCs, and to ultimately detach. We could not solve this problem by modulating the glow discharge parameters, neither by varying the time period nor by modulating the vacuum intensity. We suspect this problem to be due to inefficient coating after plasma bonding and a way to overcome that might be to use different coating materials, such as (3-aminopropyl)-triethoxysilane (Nguyen et al., 2016). Alternatively, the concentration of PLL could also be increased, but this runs the risk of clogging microgrooves, especially when PLL-coating is followed by coating with laminin.

The experimental procedure described herein is quite robust overall, but experience with handling primary neurons in general, and microfluidic devices in particular, is likely to be advantageous for its successful implementation.

We have used our novel microfluidic strategy for a series of experiments involving sensory neurons derived from DRG and TG of adult mice. We tested the neurons’ responsiveness to a selection of neurotrophic factors and neuropoietic cytokines in the primary axonal outgrowth model as well as in the IVA paradigm. Neurotrophins are presumably the most well characterized neurotrophic factors, known for their capability to not only support neuronal survival, but also to have a profound beneficial effect on axonal outgrowth and regeneration. In our experiments, we included NGF, BDNF and NT-3, which are known to address individual subtypes of sensory neurons by binding to members of the Trk family of receptor tyrosine kinases (RTKs; Wright and Snider, 1995; Gavazzi et al., 1999). Furthermore, we included GDNF, a neurotrophic factor promoting axonal growth by binding to the RTK Ret (Leclere et al., 1998), as well as CNTF, a member of the gp130 ligand family, which also contains leukemia inhibitory factor (LIF) and interleukin 6 (IL-6; Bauer et al., 2007). We opted for CNTF because in a study on gp130 ligands and their impact on axonal regeneration, this ligand had the strongest effect on phosphorylation of signal transducer and activator of transcription 3 (STAT3) and axonal elongation of adult DRG neurons (Quarta et al., 2014). The transmembrane glycoprotein gp130 is the signal-transducing constituent of various cytokine receptor complexes and has been implicated in promoting axonal regeneration after peripheral nerve injury by activating the janus kinase (JAK)/STAT signaling cascade (Bauer et al., 2007). Leptin, finally, is best known as an adipocyte-derived hormone that regulates body weight and is involved in the modulation of fat metabolism (Munzberg and Morrison, 2015). Its receptor, leptin receptor (LepR), is a member of the gp130 receptor family, and as such, also couples to the JAK/STAT pathway. There are six isoforms of LepR known, the functions of which are believed to be highly tissue- and cell type-specific (Wauman et al., 2017). LepR is expressed in sensory neurons (de Lartigue et al., 2014), and in DRG neurons from adult mice lacking gp130 leptin was demonstrated to partially rescue an axonal outgrowth phenotype, suggesting a significant role of leptin and LepR in axonal growth and regeneration (Quarta et al., 2014). By providing NGF, BDNF, NT-3, GDNF, CNTF and leptin in a single cocktail (6x cocktail), we intended to maximally induce RTK and JAK/STAT signaling, and, by comparing to simpler cocktails of trophic factors (3x cocktail comprising the neurotrophins NGF, BDNF and NT-3, or NGF only), document any additive effects on axonal elongation between these two major signaling modules.

Gradual enrichment of trophic factor cocktails resulted in continuously improved total axonal outgrowth both in the primary axonal outgrowth model as well as in the IVA paradigm indicating that BDNF and NT-3 promote axonal growth beyond NGF, an effect that in turn was topped by the additional administration of GDNF, CNTF and leptin. The results of the comparison NGF vs. 3x cocktail are at odds with a previous study conducted with adult rat DRG neurons using a Campenot chamber. While NGF was reported to have a robust growth-promoting effect, the combination of NGF with BDNF or NT-3 did not result in enhanced outgrowth and, in the case of BDNF, it even inhibited NGF-dependent growth (Kimpinski et al., 1997). This conflicting data and the general lack of studies that systematically evaluate the effects of topically applied growth factors and cytokines, and their combinations, on axonal regeneration illustrate the need for high-throughput experimentation with peripheral neurons in compartmentalized microfluidic cultures.

What we observed both in the primary outgrowth model as well as in the IVA paradigm has become known as the “priming” or “conditioning” of neurons for enhanced axonal growth. This phenomenon was originally described *in vivo* as the conditioning lesion response in the sciatic nerve, which denoted an increased rate of axonal regenerative growth if that nerve had been lesioned several days earlier (McQuarrie and Grafstein, 1973; McQuarrie, 1978; McQuarrie et al., 1978). This conditioning even allows for peripheral fibers to regenerate within the CNS, such as the dorsal column, if the central lesion is preceded by a peripheral nerve lesion (Richardson and Issa, 1984; Neumann and Woolf, 1999). It is now known that a peripheral lesion induces a broad and robust injury response by profoundly changing the neurons’ gene expression program (Smith and Skene, 1997). Initially triggered by acute signals, notably calcium waves, a signaling cascade leads to the activation of a number of transcription factors that coordinate the expression of regeneration-associated genes (RAGs), which ultimately switch the neurons into an intrinsic axonal growth state (Mar et al., 2014; Doron-Mandel et al., 2015). The most relevant signaling nodes implicated in mounting this injury response seem to be cyclic adenosine monophosphate (cAMP; Cai et al., 1999; Neumann et al., 2002), mitogen-activated protein kinases (MAP kinases; Chierzi et al., 2005; Perlson et al., 2005; Barnat et al., 2010), the phosphatidylinositol-3 kinase (PI3K)-GSK3 module (Hur et al., 2013), as well as the JAK/STAT module (Liu and Snider, 2001; Qiu et al., 2005; Bareyre et al., 2011). By binding and activating RTKs, neurotrophic factors activate both MAP kinases and PI3K (Airaksinen and Saarma, 2002; Reichardt, 2006). Moreover, neurotrophins were shown to elevate cAMP levels through inhibiting phosphodiesterase 4 (PDE4), the main enzyme responsible for cAMP hydrolysis, which causes intracellular cAMP to rise (Gao et al., 2003). gp130 cytokines are believed to be the main activators of the JAK/STAT signaling pathway following nerve injury, with a particular focus having being put on LIF and IL-6 (Zigmond, 2011). As shown recently, however, leptin through LepR also seems to contribute to STAT3 phosphorylation and axonal elongation in DRG neurons (Quarta et al., 2014). In the same study, the three gp130 ligands, LIF, IL-6 and CNTF were assessed for their potential to induce STAT3 phosphorylation and to promote axonal elongation and CNTF, unexpectedly, proved to be by far the most potent cytokine in this respect (Quarta et al., 2014). Evidently, a lot of further studies are required to determine the potency of individual growth factors to push neurons into an axonal growth state, and to delineate any additive or synergistic effects of growth factor combinations. It would be of value to streamline the 6x cocktail by deleting any redundant growth factors, or to supplement the recipe with chemical compounds that specifically interfere with distinct signaling pathways. It is exactly studies like these that will greatly benefit from the presented microfluidic approach. For reasons outlined above, experiments with peripheral neurons in modern MFCs have remained limited. In fact, to the best of our knowledge, this is the first time that the intrinsic axonal growth state of sensory neurons, not to mention sensory neurons derived from cranial ganglia, could be precisely quantified.

In conclusion, we present a simple microfluidic approach that dramatically reduces the number of neurons required for seeding a microfluidic device. Such a high level of efficiency will enable high-throughput microfluidic experimentation with DRG neurons and will open up the way to microfluidic experiments with sensory neurons derived from cranial ganglia and other neurons with similarly limited availability, such as sympathetic neurons, ciliary neurons and motor neurons. There is a particularly urgent need for a reliable and simple means to systematically evaluate the effects of topically applied growth factors and cytokines on peripheral axonal regeneration, and the approach presented here will significantly contribute to resolving this issue.

## Author Contributions

GJ performed experiments and analyzed data. SM performed experiments. MO analyzed data. RS designed the study, analyzed data and wrote the manuscript.

## Conflict of Interest Statement

The authors declare that the research was conducted in the absence of any commercial or financial relationships that could be construed as a potential conflict of interest.

## Abbreviations

BDNF: brain-derived neurotrophic factor
cAMP: cyclic adenosine monophosphate
CNS: central nervous system
CNTF: ciliary neurotrophic factor
DIV: days *in vitro*
DRG: dorsal root ganglion
IL-6: interleukin 6
IVA: *in vitro* axotomy
JAK: janus kinase
LepR: in receptor
LIF: leukemia inhibitory factor
MAP kinase: mitogen-activated protein kinase
MFC: microfluidic chamber
NGF: nerve growth factor
NT-3: neurotrophin 3
PI3K: phosphatidylinositol-3 kinase
PLL: poly-L-lysine
PNS: peripheral nervous system
ROI: region of interest
RT: room temperature
RTK: receptor tyrosine kinase
STAT3: signal transducer and activator of transcription 3
TG: trigeminal ganglion.
